# Ligand Screening of Membrane Proteins Embedded in Nanodiscs: How to Manage Non-Specific Interactions in Weak Affinity Chromatography?

**DOI:** 10.3390/molecules29122814

**Published:** 2024-06-13

**Authors:** François-Xavier Vidal, Adrien Deloche, Gabrielle Zeder-Lutz, Maria Hideux, Renaud Wagner, Vincent Dugas, Claire Demesmay

**Affiliations:** 1 Universite Claude Bernard Lyon1, Institut des Sciences Analytiques, UMR5280, CNRS, 5 rue de la Doua, 69100 Villeurbanne, Franceadrien.deloche@isa-lyon.fr (A.D.); vincent.dugas@univ-lyon1.fr (V.D.); 2 Plateforme IMPReSs, CNRS UMR7242, Biotechnologie et Signalisation Cellulaire, Ecole Supérieure de Biotechnologie de Strasbourg, 67400 Illkirch, France; gabrielle.zeder-lutz@unistra.fr (G.Z.-L.); renaud.wagner@unistra.fr (R.W.); 3 Institut de Recherche et Développement SERVIER Paris-Saclay-22, Route 128, 91190 Gif sur Yvette, France

**Keywords:** affinity chromatography, GPCR, weak affinity interactions, adenosine receptor, fragment screening, nano-liquid chromatography

## Abstract

Miniaturized weak affinity chromatography is emerging as an interesting alternative to conventional biophysical tools for performing fragment-screening studies in the context of fragment-based drug discovery. In order to push back the analytical limits, it is necessary not only to control non-specific interactions with chromatographic support, but also to adapt this methodology by comparing the results obtained on an affinity column to a control column. The work presented in this study focused on fragment screening that targets a model membrane protein, the adenosine A2A receptor, embedded in nanodiscs (NDs) as biomimetic membranes. By studying the retention behavior of test fragment mixtures on supports modified with different types of NDs, we were able to determine the contribution of ND-related non-specific interactions, in particular the electrostatic effect of anionic phospholipids and the hydrophobic effect of neutral phospholipids. Different strategies for the preparation of control columns (empty NDs, orthosteric site blocking) were investigated and are presented for the first time. With these two types of control columns, the screening enabled the identification of two new fragments of AA_2A_R, which were confirmed by competition experiments and whose K_d_ values, estimated directly during the screening or after the competition experiments in frontal mode, were in good agreement.

## 1. Introduction

In the field of fragment-based drug discovery, weak affinity chromatography (WAC) has found its place in the arsenal of biophysical methods (ITC, SPR, BLI, NMR, and nanoDSF) [1,2,3,4] for detecting weak interactions (on a micromolar (μM) to millimolar (mM) scale) between a target protein and small ligands called fragments. In weak affinity chromatography, the protein target is immobilized on the chromatographic column and a library of fragments is screened by injecting pools of fragments. The higher the affinity of the fragments for the immobilized protein, the more they will be retained. The popularity of WAC for fragment library screening is linked to its multiplexing capacity and the significant reduction in reagent consumption (protein target and fragment). In fact, the possibility of reusing chromatographic supports, combined with their miniaturization in columns with very small internal diameters (down to a few tens of microns), means that the amount of protein required can be significantly reduced (down to the µg level). In addition, the coupling of WAC with mass spectrometry detection means that a large number of fragments (pools of up to 50 or even 100 fragments) can be monitored per analysis, reducing the screening times. These advantages have been used to open up fragment screening to membrane proteins, a class of proteins that is particularly difficult to study [5,6]. In addition to the difficulty of producing and isolating large quantities of these proteins in their functional state, they are usually unstable in a non-lipidic environment. Various approaches have been proposed to apply weak affinity chromatography to membrane proteins, either by immobilizing them in their natural environment (i.e., cell membrane fragments) or by immobilizing macromolecular assemblies (biomimetic membranes) that stabilize them, such as proteolipodiscs (PEG-stabilized bilayer disks) [7], proteoliposomes [8], or, more recently, nanodiscs (NDs, apolipoprotein-stabilized lipidic nanoparticles) [9].

While the ability to detect weak interactions is primarily dependent on the number of active proteins immobilized per unit volume of the stationary phase, non-specific interactions can lead to a misestimation of the affinity. Fragments may actually interact with the chromatographic support or with components of the cellular or biomimetic membranes. These non-specific interactions are a common bottleneck in fragment screening and a source of potential false positives/negatives in the identification of hits. These interactions are not always sufficiently discussed in the literature, or are even ignored, although knowledge of the contribution of these non-specific interactions to overall fragment retention is critical.

A first strategy to take into account the non-specific interactions is to compare the retention of the fragments at a low concentration (less than 0.1 K_d_)—corresponding to specific binding—to that at a high concentration (higher than 10 K_d_)—representing the saturation point of the receptor and also corresponding to the non-specific binding [9,10]. If the retention factors are equal, there is no affinity between the fragment and the target. The greater the difference in retention, the greater the affinity of the compound for the target.

The use of control columns seems to be the best solution for estimating and accounting for non-specific interactions. This methodology is based on measuring the retention factors of the different library fragments on a control column (column without proteins or whose interaction site is blocked by a ligand). These non-specific retention factors (k_ns_) are subtracted from the retention factors measured on a column with active protein binding sites (k_tot_) to account for specific interactions only (k_spe_). For soluble proteins immobilized directly on the stationary phase, control columns are columns containing only the chromatographic support. Such control columns are not suitable for membrane proteins because they do not take into account the non-specific interactions associated with the biomimetic membrane that stabilize them in aqueous media. For membrane proteins, several solutions have been considered to correct for non-specific interactions: either the use of control columns with empty biomimetic membranes [4,7,11,12] or the use of columns with the target protein partially inactivated by an acidic treatment or blocked by a known ligand [7]. However, the correction is sometimes not fully satisfactory, demonstrating the difficulty of accounting for the contribution of non-specific interactions on the affinity column.

Recently, we have shown that the immobilization of purified native membrane proteins stabilized in NDs (self-assembled discoidal fragments of lipid bilayers 8–16 nm in diameter, stabilized in a solution by an amphipathic helical scaffold protein) in monolithic capillary columns is an interesting strategy for WAC with intact membrane proteins on a miniaturized scale [9]. In order to move from proof-of-concept to membrane protein screening, we proposed a comprehensive study of non-specific interactions on this type of affinity column to select the best type of control column. Here, we applied a previously established methodology [13] to assess the contribution of NDs themselves to non-specific interactions through the evaluation of different control columns, functionalized either with empty NDs or with NDs containing a model G protein-coupled receptor (GPCR), the adenosine A_2A_ receptor (AA_2A_R), whose specific binding site is blocked by a ligand.

## 2. Results and Discussion

**Figure 1:**
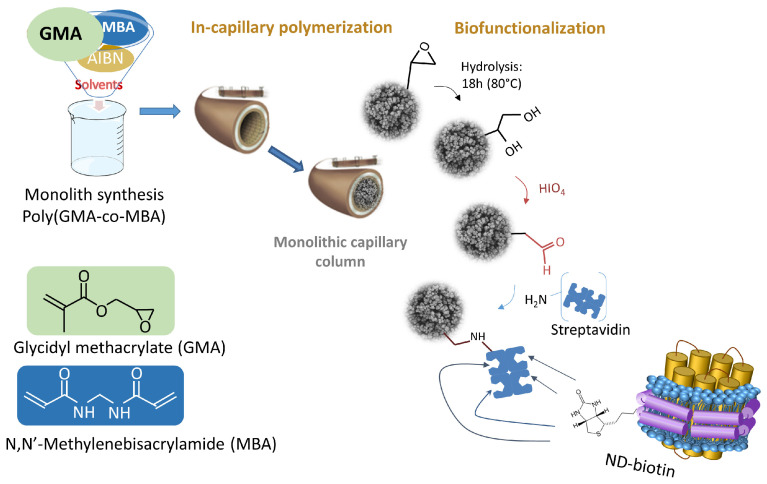
Schematic representation of the in-capillary poly(GMA-co-MBA) synthesis, surface functionalization, and immobilization of biotinylated nanodiscs.

Affinity columns were synthesized as shown in Figure 1. A poly-GMA-co-MBA monolith was synthesized in situ in a 75 µm i.d. capillary. After the epoxy-ring opening, diol oxidation into aldehyde streptavidin was immobilized according to the Schiff base method. The streptavidin-generic column was further modified by percolating the biotinylated-ND solution.

### 2.1. Influence of NDs on Non-Specific Retention

**Figure 2:**
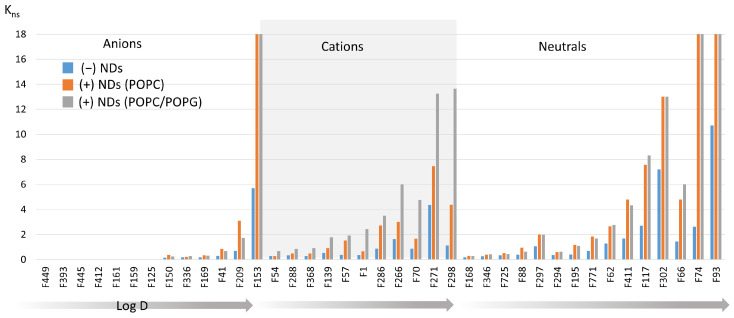
Plot of the non-specific retention factor k_ns_ of the 39 fragments on poly(GMA-co-MBA) monoliths in the absence (−) and presence (+) of NDs, measured in 20 mM mobile-phase ammonium acetate (pH of 7.4). The fragments were classified by their charge state and ordered by increasing logD values (pH of 7.4).

The effect of the presence of NDs on the support was evaluated by following the methodology previously developed to evaluate and characterize the non-specific interactions of fragments on the monolithic support itself [13]. The same selected set of 39 fragments was analyzed on the monolithic poly(GMA-co-MBA) support in the absence ((−) NDs) and in the presence of empty NDs, prepared with either zwitterionic phospholipid phosphatidylcholine ((+) NDs (POPC)), or a mixture of POPC and negatively charged phospholipid phosphatidylglycerol ((+) NDs (POPC/POPG)). These fragments were selected from a library of fragment-like molecules that covered the whole range of physico-chemical properties in terms of their net charge, logD, and H-bond donor and acceptor. The physical–chemical properties and chemical structures of the set of fragments are detailed in Table S1. The total number of NDs was the same for all columns (B_tot_ = 28.6 ± 0.5 pmol/column). The retention factors k_ns_ measured for each fragment on the three supports are compared in Figure 2.

The retention factors measured on the monolithic support with NDs ((+) NDs) were always higher than those measured on the supports without NDs ((−) NDs), regardless of the fragment and the phospholipid type. This result shows the significant contribution of NDs to non-specific interactions. Furthermore, the increase in retention factors seemed to be strongly correlated with the logD at a pH of 7.4 for the fragments, regardless of their charge state. This increase in non-specific interactions was, therefore, mainly due to hydrophobic effects. Moreover, the comparison of the results observed on the supports with the NDs prepared with the neutral POPC or containing a fraction of negatively charged POPG showed a different behavior for the neutral and charged fragments. On the one hand, the difference in retention factors between the two types of functionalized supports (POPC and POPC/POPG) was not significant for neutral fragments. On the other hand, the retention factors increased for most of the cationic fragments, while they decreased for the anionic compounds, except for F288 and F266, when the NDs contained POPG. This behavior can be attributed to electrostatic effects caused by the charges carried by POPG, i.e., an attractive effect for cationic compounds and a repulsive effect for anionic ones. To validate this assumption, the retention factors on the NDs (POPC/POPG) were measured using a mobile phase with a high ionic strength (160 mM ammonium acetate solution with a pH of 7.4) designed to shield electrostatic interactions. At such an ionic strength, the retention factors of all the fragments on the POPC/POPG NDs were not significantly different from those observed on the POPC NDs.The influence of NDs and their composition on the non-specific retention of fragments therefore highlights the importance of the choice of the control column to extract the corresponding non-specific signal from the retentions obtained on affinity columns in the presence of the assayed protein receptor.

### 2.2. Evaluation of Different Types of Control Columns

For screening purposes, selecting the best control column is critical for limiting the number of false positives or negatives. Different types of control columns were evaluated and compared on the basis of their ability to identify hits in non-ligand mixtures.

In order to limit the number of experiments to be performed and to facilitate the comparison of these different approaches, the number of evaluated fragments was reduced to nine, including three neutral fragments (F195, F294, and F297), three cationic fragments (F1, F139, and F70), and three anionic fragments (F169, F209, and F41). These fragments were selected as the best non-specific interaction reporters because of their strong interactions with the NDs on the support. Besides this mixture of nine non-ligands, three known ligands of AA_2A_R were also added as positive controls. The retention factors of these 12 fragments measured on the different control columns (k_ns_) were then compared to their retention factors on affinity columns (+AA_2A_R) (k_affinity_). These data were further used to rank the control columns: in an ideal case, the difference in retention factors between the affinity and control columns should be as small as possible for the non-ligands. Similarly, the retention on the affinity columns should be higher than that on the control columns for known ligands.

Finally, as in the first series of experiments, the same amount of NDs was immobilized (about B_tot_ = 28.6 ± 0.5 pmol/column) for all the columns (affinity and control columns).

#### 2.2.1. Evaluation of Control Columns with Empty NDs

Empty-ND control columns (−AA_2A_R) and affinity-ND columns (+AA_2A_R) were first prepared with POPC, since this zwitterionic phospholipid limits the non-specific retention regardless of the fragments studied. Mixtures containing the 12 fragments (9 non-ligands and 3 known ligands of AA_2A_R) were injected on both columns and the retention factors were compared.

**Figure 3:**
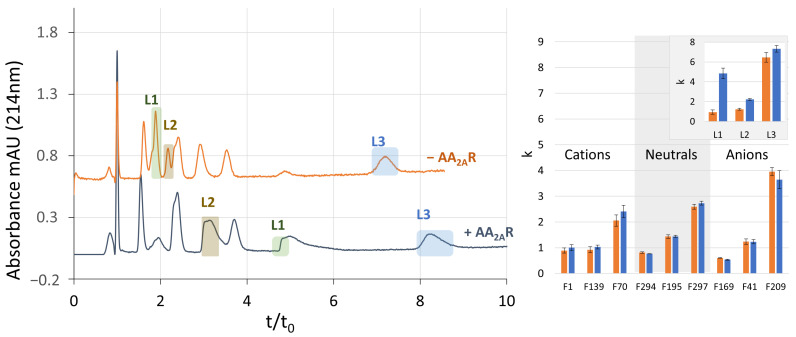
Evaluation of empty NDs (POPC) as control columns. (**Left**): Chromatograms of the set of 12 fragments (9 non-ligands and 3 ligands) injected on the empty-ND (POPC)-functionalized column (−AA_2A_R) and the affinity-ND (POPC) column (+AA_2A_R). Column length, 8.5 cm; mobile phase, 20 mM ammonium acetate (pH of 7.4); fragment concentration, 50 µM. (**Right**): Graphs showing the variation in the retention factors on the empty (orange) and affinity (blue) columns for the 9 non-ligands and the 3 known ligands of AA_2A_R (insert, L1, L2, and L3). Error bars are the standard deviation calculated from three repetitions.

Figure 3 (left) compares the separations of the 12 fragments obtained on control columns with empty NDs (−AA_2A_R) and on affinity columns (+AA_2A_R)). The retention times of the fragments used as non-ligands did not vary significantly between the two columns. Accordingly, the calculated retention factors (Figure 3, right) were also not significantly different. On the other hand, the retention times and the retention factors of the known ligands (L1, L2, and L3) increased significantly on the AA_2A_R affinity column, as expected. These results suggest that a column with empty NDs (POPC) may constitute a well-suited control column.

**Figure 4:**
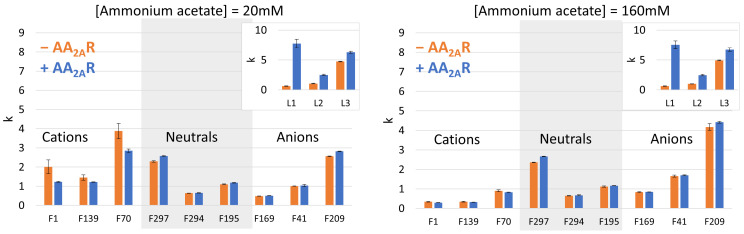
Bar charts comparing the retention factors measured on columns functionalized with POPC/POPG NDs with (+) and without (−) AA_2A_R for ligand (insert, L1, L2, and L3) and non-ligand fragments. Experiments were performed either in 20 mM mobile-phase ammonium acetate with a pH of 7.4 (**left**) or in 160 mM mobile-phase ammonium acetate with a pH of 7.4 (**right**).

The same set of experiments was then implemented with NDs prepared with a mixture of zwitterionic and anionic phospholipids (POPC/POPG). The retention factors calculated from the resulting chromatograms are presented in Figure 4.

The 20 mM ammonium acetate mobile phase was first selected for its compatibility with mass spectrometric detection. With this low-ionic-strength mobile phase, the differences in the retention factors between the control and affinity columns were significant for cationic non-ligands, with a higher retention on the control column than on the affinity column (Figure 4, left). This can be attributed to the reduced electrostatic interactions of cationic fragments with the POPG anionic phospholipids in (+AA_2A_R) NDs, since the AA_2A_R protein occupies a space that is filled by phospholipids in empty NDs. In a screening context, a decrease in the retention on the affinity column could lead to false negatives if the affinity of a fragment is compensated for by a decrease in non-specific electrostatic interactions. Therefore, a column with empty NDs (POPC/POPG) cannot be used as a control column for cationic compounds with a low-ionic-strength mobile phase. As shown in Figure 4 in the right panel, screening in a higher-ionic-strength mobile phase allows the contribution of non-specific electrostatic interactions to be limited by charge screening. A column with empty NDs (POPC/POPG) can, therefore, be used as a control column under this condition for all types of compounds. Such a high-ionic-strength mobile phase is, however, not well suited for MS detection.

Control columns with empty NDs seem to be a satisfactory solution that is simple and universal. Only one single screening of the fragment library on such a control column is actually required to determine the non-specific retention factors, which can then be subtracted from the apparent retention factors measured on any affinity column. The only requirement is a high reproducibility in the affinity column preparation.

#### 2.2.2. Evaluation of Control Columns in Which the Orthosteric Protein Binding Site Is Blocked

**Figure 5:**
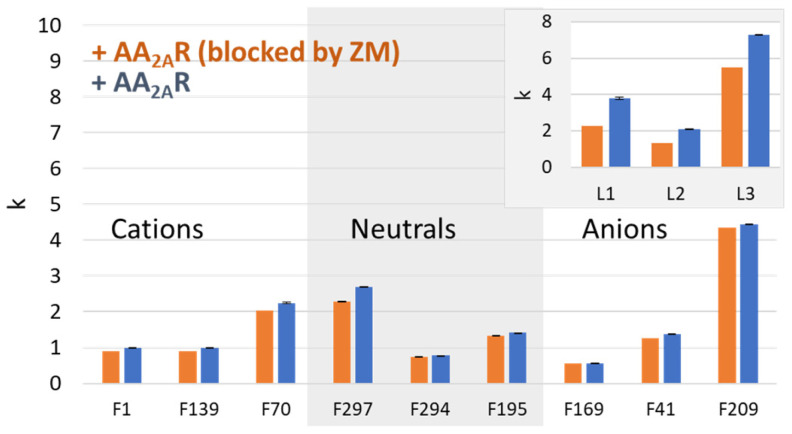
Bar charts comparing the retention factors of the 12 test fragments measured on columns functionalized with NDs (POPC) with the (+AA_2A_R) receptor and on the same column after saturation of the membrane protein by ZM241385. The experiments were carried out in 20 mM mobile-phase ammonium acetate with a pH of 7.4.

As an alternative strategy to appraise non-specific interactions of fragments with an immobilized receptor, the present approach consists of comparing affinity columns (+AA_2A_R NDs) in which the orthosteric binding site is free (for identifying specific and non-specific interactions) or blocked by a known ligand (for determining non-specific interactions). The major advantage of such a strategy is that it eliminates any (even minor) variability due to column preparation. In these experiments, we considered saturating the orthosteric interaction site of AA_2A_R with a high-affinity ZM241385 ligand (ZM, K_d_ = 0.75 ± 0.08 nM) [14]. Using the previously described mixture of 12 fragments, the retention factors of the AA_2A_R affinity columns were then compared before and after blocking the orthosteric site with ZM (Figure 5).

The retention factors measured for most of the non-ligands (eight out of nine) were not significantly different before and after column deactivation (variation of less than 15%), indicating that blocking the orthosteric site of the protein with ZM provides a satisfactory estimate of non-specific interactions and makes it possible to discriminate ligands from non-ligands. However, this approach requires the availability of at least one high-affinity or covalent ligand, which is not the case for many receptors. In addition, a second screening must be performed on each affinity column to identify the retention time shifts associated with specific interactions. Another drawback associated with this type of column control is that any compound interacting with potential allosteric sites is then considered a false negative.

### 2.3. Nano-WAC Screening Using Empty NDs (POPC) and ZM-Blocked Control Columns

As a proof-of-concept, a screening of the set of 39 fragments was then performed with empty NDs (POPC) or ZM-blocked AA_2A_R NDs as a control column. As explained earlier in this article, this set of fragments can be considered representative of a fragment library, as the fragments cover the whole range of physico-chemical properties in terms of their net charge, logD, and H-bond donor and acceptor. To be identified as a ligand, the retention factor of a fragment should increase by at least 15% (the inter-column variation coefficients of retention factors range from a few to 10% for all fragments) with a minimum threshold increase of 0.5. With such a threshold of 0.5, fragments with K_d_ values as high as 250 µM should be detected (with respect to an active site volumetric density of 33 pmol/300 nL; see Supplementary Figure S1 for its determination by frontal affinity chromatography with a known ligand). Lowering this threshold to 0.25 could extend the K_d_ range to weaker affinities (K_d_ values as low as 500 µM) at the expense of a higher risk of false positives. These results are summarized in Table 1, where the identified ligands are highlighted in green. With a retention factor variation threshold of 0.5 and a minimum retention factor increase of 15%, five fragments were identified as ligands. Three of these five fragments were the known AA_2A_R ligands (caffeine, F462, and F411). Two other fragments (F725 and F271) were identified as new potential ligands. The magnitude of the observed shift in the retention between the affinity and control columns can be used to estimate the K_d_ of ligands according to Equation (1), where B_act_/V_m_ is the volumetric density of active binding sites (110 pmol/µL) that was determined with caffeine as a ligand with a known K_d_ value. For low-affinity ligands (high K_d_ value) and a low ligand concentration (due to chromatographic dilution), the ligand concentration can be neglected before the K_d_ value.

(1) $$k=\frac{1}{K_{d}+[L]}*\frac{B_{act}}{V_{m}}$$

In this way, K_d_ values of approximately 50 and 200 µM were determined for F271 and F725, respectively.

### 2.4. Confirmation of the Hits and Evaluation of Their Affinity for AA_2A_R by Nano-FAC Experiments

**Figure 6:**
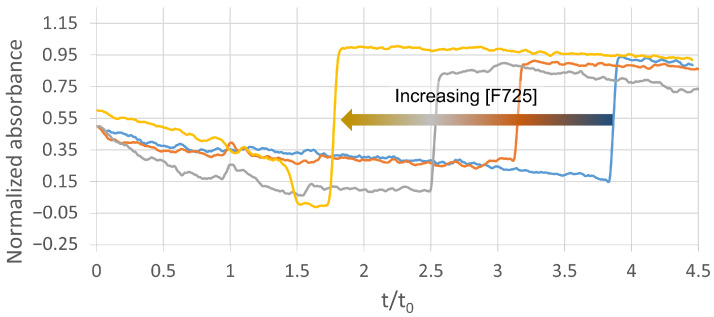
Frontal affinity chromatograms of caffeine (25 µM) on affinity column (+A2A) in absence (blue plot) and in presence of increasing concentrations of competitor (F725): 200 µM (orange plot), 500 µM (grey plot), and 2 mM (yellow plot). Experiment carried out in 20 mM mobile-phase ammonium acetate with pH of 7.4. UV detection occurred at 260 nm.

Frontal affinity chromatography (FAC) on an AA_2A_R-affinity column was used to confirm the hits and further investigate the affinity of these two potential fragments. FAC experiments were conducted using competition experiments, which monitor the extent to which an unknown fragment displaces an indicator (a ligand with a known affinity for a known protein binding site) for a particular target. Caffeine, with a K_d_ value of 25 µM, was used as an indicator ligand. Figure 6 illustrates the shift in the breakthrough time of caffeine with an increasing concentration of F725 in the mobile phase. This relative shift of the caffeine “indicator” allowed the dissociation constant of the unknown fragment (K_d, fragment_) to be estimated according to Equation (2).

(2) $$K_{d,fragment}=\frac{K_{d,caffeine}\times \left[fragment\right]}{\frac{B_{act}}{\left(V_{r}-V_{m}\right)}-K_{d,caffeine}-\left[caffeine\right]}$$

where B_act_ is the number of AA_2A_R binding sites on the column (29 pmol) and Vr and Vm are the retention and hold-up volume. The decrease in the breakthrough time of caffeine (Figure 6) with an increasing concentration of the fragment F725 confirmed that the two fragments share the same AA_2A_R binding site. The situation was similar for F271. The apparent K_d,fragment_ values obtained from Equation (2) were estimated to be 30 ± 6 µM and 200 ± 10 for F271 and F725, respectively. It should be noted that these K_d_ values are well correlated with the K_d_ values estimated during the screening step. Furthermore, the criteria used to discriminate specific ligands from non-ligands seemed to be relevant and validated the analytical workflow, regardless of the column control used. Here, two new fragments were detected as specific ligands of AA_2A_R.

## 3. Materials and Methods

### 3.1. Reagents and Buffers

The (3-methacryloxypropyl)-trimethoxysilane (γ-MAPS); glycidyl methacrylate (GMA); acrylamide; N,N′-methylenebis(acrylamide) (MBA); dodecanol; 1,4-butanediol; dimethyl sulfoxide (DMSO); sodium periodate; lithium hydroxide; dipotassium hydrogen phosphate (K_2_HPO_4_); o-phosphoric acid; ammonium acetate; sodium cyanoborohydride; triethylamine (TEA); azobis(isobutyronitrile) (AIBN); streptavidin (from Streptomyces avidinii, affinity-purified, ≥13 U mg^−1^ of protein); and ligands (Table S1) were purchased from Sigma-Aldrich (L’Isle d’Abeau, France). All the aqueous solutions were prepared using >18 MΩ of deionized water. A 67 mM phosphate buffer was prepared by dissolving 1.17 g of K_2_HPO_4_ in 100 mL of ultrapure water, and the pH was adjusted to 7.4 with phosphoric acid. An acetate buffer was prepared by dissolving 1.54 g of 20 mM ammonium acetate or 12.3 g of 160 mM ammonium acetate in 100 mL of ultrapure water, and the pH was adjusted to 7.4 with acetic acid.

### 3.2. Synthesis of Poly(GMA-co-MBA) Monolithic Capillary Columns

A total of 75 µm ID, 375 µm OD, fused-silica capillaries with a polyimide coating (TSP) were purchased from Cluzeau Info Labo (Sainte-Foy-La-Grande, France). Capillaries with a length of 15 cm were activated by flushing a 5% (*v*/*v*) solution of γ-MAPS in methanol/water (95/5, *v*/*v*) and 2.5% TEA for 1 h at 7 bars. The capillaries were then rinsed with methanol for 15 min at 7 bars and dried at room temperature under a nitrogen stream before use.

The poly(GMA-co-MBA) monoliths [1] were prepared by mixing 3330 mg of DMSO, 1480 mg of 1,4-butanediol, 1850 mg of dodecanol, 320 mg of MBA, and 480 mg of GMA for 1 h at room temperature. A total of 8 mg of AIBN was added, and the final mixture was sonicated for 15 min at room temperature. The activated capillary was then filled with the polymerization mixture under 1 bar of N2 pressure and the ends of the capillary were sealed. The polymerization reaction was performed in a water bath at 57 °C for 18 h. After polymerization, the monoliths were rinsed with methanol for 1 h and kept wet until use.

### 3.3. Column Biofunctionalization

#### 3.3.1. Preparation of Streptavidin-Functionalized Monolithic Capillary Columns

The poly(GMA-co-MBA) monoliths were first hydrolyzed into diols in hot water at 80 °C for 18 h and then subjected to a 0.12 M NaIO4 solution at a pH of 5.5 for 1 h at 7 bars to oxidize the diol groups into reactive aldehyde ones. Streptavidin was immobilized (Schiff base method) by percolating a 1 mg mL^−1^ streptavidin solution and a 4 mg mL^−1^ NaBH_3_CN solution in 67 mM of phosphate buffer (pH of 6) through the column for 18 h at 7 bars at room temperature. At the end of the immobilization step, the columns were flushed with sodium borohydride (2.5 mg mL^−1^ of phosphate buffer, 67 mM, pH of 8, for 2 h at 7 bars) to reduce the residual aldehydes. The streptavidin columns were rinsed with phosphate buffer and stored at 4 °C.

#### 3.3.2. Preparation of ND-Functionalized Monolithic Capillary Columns

The control and affinity columns were prepared by percolating empty NDs or NDs incorporating the AA_2A_R receptor. Biotinylated-ND solutions (µM range) were percolated through the streptavidin columns with in situ UV monitoring at 280 nm to stop the sample flow when saturation was reached. This allowed the quantification of the total amount of NDs captured (B_tot_).

### 3.4. Preparation of Nanodisc Samples

#### 3.4.1. Production and Purification of AA_2A_R

A recombinant his-tagged human A_2_A receptor was heterologously produced with the yeast *Pichia pastoris* and further purified as previously described [15]. Briefly, the produced AA_2A_R receptor was extracted from whole-membrane fractions with a solubilization buffer (50 mM HEPES, pH of 7.4; 500 mM NaCl; 0.5% n-dodecyl-β-d-maltopyranoside (β-DDM) (*w*/*v*); 0.05%cholesteryl hemisuccinate (CHS) (*w*/*v*); 30 mM imidazole; 1 µM 1,3-dipropyl-8-cyclopentylxanthine (DPCPX); 0.3 mM EDTA; and one antiprotease tablet) prior to purification on an IMAC column (1 mL HisTrap HP column, Cytiva, Marlborough, MA, USA) in a buffer comprising 50 mM HEPES (pH of 7.4), 500 mM NaCl, 0.05% β-DDM (*w*/*v*), 0.005% CHS (*w*/*v*), 100 mM imidazole, and 1 µM DPCPX. The resulting fractions were pooled and injected onto a HiLoad Superdex 200 Increase 16/600 PG column (Cytiva, Marlborough, MA, USA) with a buffer comprising 50 mM HEPES (pH of 7.4), 150 mM NaCl, 0.02% β-DDM (*w*/*v*), 0.002% CHS (*w*/*v*), and 1 µM DPCPX. The fractions corresponding to the monomeric AA_2A_R receptor in detergent were pooled, concentrated to about 0.8 mg/mL on a Vivaspin20 30K MWCO deviceβ—(Sartorius, Bangkok, Thailand), and directly used for reconstitution into lipid nanodiscs.

#### 3.4.2. Membrane Scaffold Protein (MSP) Purification and Biotinylation

The membrane scaffold protein MSP1E3D1(−) was produced and purified from *E. coli* according to [16]. The MSP1E3D1(−) was further biotinylated with the Thermo Scientific reagent EZ-Link^TM^ NHS-PEG_4_-Biotin (Waltham, MA, USA), as previously described [7], and finally purified on a HiTrap desalting column (Cytiva) in a storage buffer comprising 20 mM Tris-HCl (pH of 7.4), 100 mM NaCl, and 0.5 mM EDTA. After a snap-freezing step in liquid nitrogen, the biotinylated MSP1E3D1(−) was stored at −80 °C until use.

#### 3.4.3. Assembly and Purification of Empty NDs and AA_2A_R NDs

The biotinylated MSP1E3D1(−) was mixed at a 1:70 molar ratio with purified lipids dissolved at 24 mM in a buffer comprising 50 mM HEPES (pH of 7.4), 150 mM NaCl, and 48 mM sodium cholate, with either POPC alone for the NDs (POPC) or a mixture of POPC/POPG at a 3/2 molar ratio for the NDs (POPC/POPG). The MSP/lipid mixtures were incubated for 15 min on ice before the detergent-purified receptor was added at a 1:10 AA_2A_R:MSP1E3D1(−) molar ratio and incubated for a further 60 min on ice. Self-assembly was initiated and amplified by detergent removal using BioBeads SM-2 (Biorad, Hercules, CA, USA) added at 0.25 g per mL of reconstitution mixture. After incubation overnight at 4 °C on a tube rotator, the Biobeads were removed by centrifugation, and the recovered supernatants were directly injected into a 1 mL HisTrap HP column (Cytiva, Marlborough, MA, USA) previously equilibrated in a buffer comprising 50 mM HEPES (pH of 7.4), 300 mM NaCl, and 10 mM imidazole. The flow-through fractions mainly containing empty NDs were collected and set apart for a further size exclusion purification. After extensive washing with the equilibration buffer, the fractions containing the AA_2A_R NDs were eluted in the presence of 500 mM imidazole and pooled. The two types of samples issued from the IMAC step (empty-ND and AA_2A_R-ND fractions) were then injected into a Superdex 200 Increase 10/300 GL column (Cytiva, Marlborough, MA, USA) previously equilibrated in a buffer comprising 50 mM HEPES (pH of 7.4) and 150 mM NaCl. Elution was performed at 0.3 mL.min^−1^, and the fractions containing the purified empty NDs (POPC), AA_2A_R NDs (POPC), empty NDs (POPC/POPG), or AA_2A_R NDs (POPC/POPG) were finally aliquoted, snap-frozen in liquid nitrogen, and stored at −80 °C until use.

### 3.5. Instrumentation: Nano-LC Experiments

The nano-liquid chromatography experiments were carried out with a 7100 capillary electrophoresis Agilent system (Agilent Technologies, Waldbronn, Germany) using the external pressure module (up to 12 bars) to flow mobile phases and the OpenLab CDS C.01.08 software (Agilent) to drive the instrument. All the experiments were carried out in the “short-end” injection mode, with the inlet of the capillary immersed in the solution to be injected/infused. For in situ UV detection, a window was created by burning away the polyimide coating. The analyses were all carried out at a controlled room temperature of 25 °C. The samples were hydrodynamically injected by applying a pressure at the inlet of the capillary (12 bars, 2 s). The mixture of 3 ligands (caffeine, F411, and F462) and 9 non-ligands (3 neutral fragments (F195, F294, and F297), 3 cationic fragments (F1, F139, and F70), and 3 anionic fragments (F169, F209, and F41)) was prepared at 200 µM each in a 20 mM acetate buffer.

## 4. Conclusions

This work investigated the influence of NDs (POPC or POPC/POPG) on the non-specific retention of fragments in affinity chromatography. Non-specific interactions with NDs were strongly correlated with the logD of the fragments, regardless of their charge state. For anionic NDs (prepared with a POPC/POPG mixture), the electrostatic interactions between cationic fragments and the anionic head of the phospholipid strongly increased these non-specific interactions, unless a high-ionic mobile phase shielded them. In order to take such non-specific interactions into account during the screening process, several types of control columns were considered. The use of empty NDs should facilitate a screening campaign by serving as a reference for the non-specific interactions of a library on one type of ND, which can be compared with NDs containing the target protein. The presence of charged phospholipids can lead to interpretation errors, but this can be counteracted by increasing the ionic strength of the mobile phase. However, such an increase in the mobile-phase ionic strength is incompatible with mass spectrometric detection. Therefore, if possible and depending on the protein requirement, the preparation of NDs with only neutral phospholipids (POPC) avoids this problem and opens possibilities for coupling nano-WAC to mass spectrometry. When a specific phospholipid environment is required, the use of a high-affinity or covalent ligand is a viable solution for estimating non-specific interactions. The use of these two types of control columns was considered for a screening of 39 fragments representative of a fragment library, i.e., covering the whole range of physico-chemical properties in terms of net charge, logD, and H-bond donor and acceptor. This screening enabled the identification of two new fragments of AA_2A_R, which were confirmed by competition experiments and whose K_d_ values, estimated directly during the screening or after the competition experiments in frontal mode, were in good agreement.

## Figures and Tables

**Table 1:** List of fragments screened on AA_2A_R-affinity monolithic columns using both control columns. Known ligands are written in red and detected ligands are highlighted in green.

Empty NDs		ZM-Blocked Column	
F378	F336	F378	F336
F302	F294	F302	F294
F153	F366	F153	F366
F449	F150	F449	F150
F159	F462	F159	F462
F125	F70	F125	F70
F161	F169	F161	F169
F445	F66	F445	F66
F54	F195	F54	F195
F393	F41	F393	F41
F368	F62	F368	F62
F168	F297	F168	F297
F288	F286	F288	F286
F139	F271	F139	F271
F1	F298	F1	F298
F346	F209	F346	F209
F57	F411	F57	F411
F725	F117	F725	F117
caffeine	F74	caffeine	F74
F88		F88	

## Data Availability

Data are contained within the article.

## Supplementary Materials

The following supporting information can be downloaded at: https://www.mdpi.com/article/10.3390/molecules29122814/s1. Table S1: A list of physico-chemical properties of the test substances for the evaluation of non-specific interactions on monolithic columns. Figure S1: Frontal affinity chromatography experiments for the determination of Bact (number of active binding sites) and the Kd value of caffeine as a known ligand of AA2AR.
