# Supplementary material for: Ligand Screening of Membrane Proteins Embedded in Nanodiscs: How to Manage Non-Specific Interactions in Weak Affinity Chromatography?
Source: Molecules. 2024 Jun 13;29(12):2814. doi: 10.3390/molecules29122814 (PMC11207011; doi:10.3390/molecules29122814)
Supplement: Supplementary file 1 [file molecules-29-02814-s001.zip › molecules-3033162-supplementary.pdf]

Table S1 :

Physicochemical properties of tests solutes

Fragment	Name	Cas Number	log D (pH 7.4)	Charge	H-Bond Donor Count (PubChem)	H-Bond Acceptor Count (PubChem)
1	Benzylamine	100-46-9	-0.56	cationic	1	1
41	5-Phenyl-2-furoic acid	52938-97-3	-0.35	anionic	1	3
54	4-(Aminomethyl)pyridine	3731-53-1	-1.3	cationic	1	2
57	Histamine	51-45-6	-2.71	cationic	2	2
62	2-Phenylimidazole	670-96-2	1.64	neutral	1	1
66	1,1,4,7,10,10-Hexamethyltriethylenetetramine	4856-97-7	0.57	neutral	2	2
70	4-(2-Keto-1-benzimidazolinyl)piperidine	20662-53-7	-1.13	cationic	2	2
74	2,2'-(Ethane-1,2-diyl)dianiline	34124-14-6	1.99	neutral	2	2
88	Thieno[3,2-b]pyridin-7-ol	107818-20-2	-0.34	neutral	1	3
93	4,4'-Dihydroxybiphenyl	92-88-6	2.26	neutral	2	2
117	2-Amino-3-benzyloxypyridine	24016-03-3	1.53	neutral	1	3
125	1-Methyl-2-pyrrolecarboxylic acid	6973-60-0	-1.86	anionic	1	2
139	2-Phenyl-2-imidazoline	936-49-2	-0.59	cationic	1	1
150	Salicylic acid	69-72-7	-0.77	anionic	2	3
153	2,4-Dihydroxybenzophenone	131-56-6	3.16	anionic	2	3
159	3-Furoic acid	488-93-7	-1.97	anionic	1	3
161	Sulfacetamide	144-80-9	-2.17	anionic	2	4
168	3-Cyano-6-methyl-2(1H)-pyridinone	4241-27-4	-0.29	neutral	1	2
169	Sulfabenzamide	127-71-9	-0.62	anionic	2	4
195	1-Benzoylpiperidine	776-75-0	1.95	neutral	0	1
209	Biphenyl-4-carboxylic acid	92-92-2	0.67	anionic	1	2
266	Benzhydrylamine	91-00-9	1.65	cationic	1	1
271	1,2,3,4-Tetrahydro-9-aminoacridine	321-64-2	0.89	cationic	1	2
286	(1R,2S)-(−)-2-Amino-1,2-diphenylethanol	23190-16-1	-0.08	cationic	2	2
288	2,3-Diaminopyridine	452-58-4	0.12	cationic	2	3
294	1-(1H-Pyrrol-2-yl)ethanone	1072-83-9	0.99	neutral	1	1
297	5-Amino-1-phenylpyrazole-4-carboxamide	50427-77-5	0.83	neutral	2	3
298	1-Phenyl-1,2,3,4-tetrahydropyrrolo[1,2-a]pyrazine	112758-89-1	2.53	cationic	1	1
302	4-Aminobiphenyl	92-67-1	2.71	neutral	1	1
336	trans-Ferulic acid	537-98-4	-1.38	anionic	2	4
346	2-Thiouracil	141-90-2	-3.6	neutral	2	2
366	3-Amino-2-chloropyridine	6298-19-7	0.97	neutral	1	2
368	4-Aminopyridine	504-24-5	-2.41	cationic	1	2
378	2,6-Dichloropyridine	2402-78-0	2.1	neutral	0	1
393	1-(4-Sulfophenyl)-3-methyl-5-pyrazolone	89-36-1	-4.31	anionic	1	5
411	Ethyl 5-amino-1-phenyl-4-pyrazolecarboxylate	16078-71-0	2.79	neutral	1	4
412	2-(carboxymethylthio)-4-methylpyrimidine	46118-95-0	-3.15	anionic	1	5
445	4-Aminohippuric acid	61-78-9	-4	anionic	3	4
449	Orotic acid	65-86-1	-4.75	anionic	3	4
725	Adenine	73-24-5	-2.06	neutral	2	4
771	4-Chlorophenyl methyl sulfone	98-57-7	1.45	neutral	0	2

Figure S1 :

Nano-FAC experiments were used to determine the quantity of ligand capture at different ligand concentrations ($q_{captured}$). In staircase method, increasing concentrations of ligand were continuously injected into the nanocolumn (Figure S1 left). This allows to determine the cumulative captured quantities ($q_{captured\ cumul.}$) of ligand at each concentration step (n).

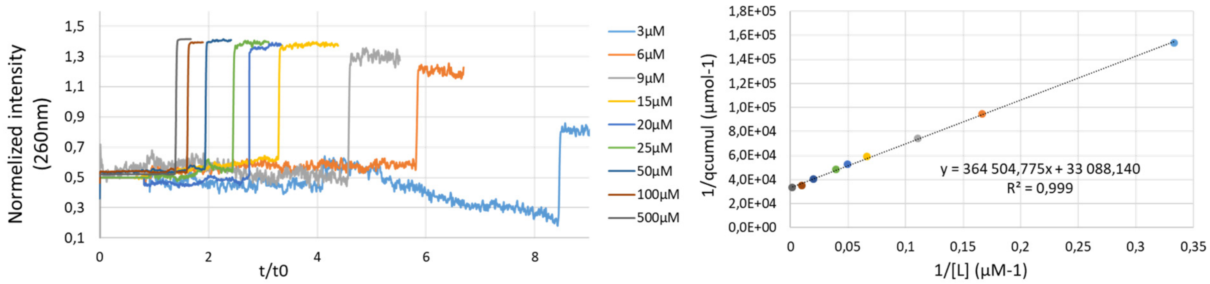

Figure S1. Frontal affinity chromatography experiments implemented to study the interaction between caffeine and immobilized $AA_{A2}R$. Caffeine solutions (solubilized in acetate buffer 20mM pH=7.4) are percolated at increasing concentrations ranging from 3 to 500µM on a poly(GMA-co-MBA) monolith column functionalized with NDs; . Column dimension : l = 8.2 cm, i.d = 75 µM ; applied pressure 12 MPa (0.14 $cm.s^{-1}$). A) Breakthrough curves of caffeine at different concentrations in ammonium acetate buffer (20mM, pH 7.4); UV detection at 260 nm; small injection of DMSO 0,1% in water was used to mark the dead time. B) Plot of the reciprocal of the quantity of the specific captured quantity of caffeine minus the non specific captured quantity of caffeine (1/(cumulQcapt - (KNS*[caffeine])) (μmol^{-1}) versus the reciprocal of caffeine concentration (1/[caffeine]) ($L.mol^{-1}$)

The plot of $\mathbf{1/q_{Cumul\ (n)}}$ vs 1/[L]n is linear if only specific interaction is observed. Presence of non-specific interactions contribution is observable as a deviation from linearity in the high concentration zone of the plot.
To take into account non-specific interactions (if they occur), it must be considered that $\mathbf{q_{Cumul\ captured\ (n)}}$ is the sum of the amount of ligand captured by specific interactions $\frac{\mathbf{B_{act}\times[L]_n}}{\mathbf{K_d+[L]_n}}$ and the amount of ligand captured by non-specific interactions $\mathbf{K_{non-specific}} \times \mathbf{[L]_n}$, leading to equation S1:

$$\mathbf{q_{Cumul\ captured\ (n)}} = \frac{\mathbf{B_{act}\times[L]_n}}{\mathbf{K_d+[L]_n}} + \mathbf{K_{non-specific}} \times \mathbf{[L]_n} \qquad \text{(Equation S1)}$$

Supposing that Knon-specific is constant, equation S1 can be written as follows :

$$\frac{\mathbf{1}}{\mathbf{q_{captured}-K_{non-specific}\times[L]_n}} = \frac{\mathbf{K_d}}{\mathbf{B_{act}}} \times \frac{\mathbf{1}}{\mathbf{[L]_n}} + \frac{\mathbf{1}}{\mathbf{B_{act}}} \qquad \text{(Equation S2)}$$

The excel solver function is used to compute the $K_{non\text{-}specific}$ factor for which the best r^2 value is reached.
In case of presence of non-specific interaction, to determine the number of active site (B_{act}) and dissociation constant properly the plot of 1/[q cumul Capt (n) − (K_{NS} x [L]n)] vs 1/[L]n was used (figure S1 B right).
The amount of binding protein sites is determined by frontal affinity chromatography (Staircase) with the caffeine and it is about 30 ± 1 pmol with K_d for the caffeine equal to 11 ± 1 µM wich is the same order with the K_d in le literature.
